# The Real World Mental Health Needs of Heart Failure Patients Are Not Reflected by the Depression Randomized Controlled Trial Evidence

**DOI:** 10.1371/journal.pone.0085928

**Published:** 2014-01-24

**Authors:** Phillip J. Tully, Gary Wittert, Terina Selkow, Harald Baumeister

**Affiliations:** 1 Department of Rehabilitation Psychology and Psychotherapy, Institute of Psychology, University of Freiburg, Freiburg, Germany; 2 Medical Psychology and Medical Sociology, Medical Faculty, University of Freiburg, Freiburg, Germany; 3 Freemasons Foundation Centre for Men’s Health, Discipline of Medicine, School of Medicine, The University of Adelaide, Adelaide, Australia; 4 Heart Failure Support Service, The Queen Elizabeth Hospital, Woodville, Australia; University of Glasgow, United Kingdom

## Abstract

**Introduction:**

International depression screening guidelines in heart failure (HF) are partly based on depression treatment efficacy from randomized controlled trials (RCTs). Our aim was to test the external validity of depression RCT criteria in a sample of real-world HF patients.

**Methods:**

HF patients admitted to 3 hospitals in South Australia were referred to a HF psychologist if not already receiving current psychiatric management by psychologist or psychiatrist elsewhere. Screening and referral protocol consisted of the following; (a). Patient Health Questionnaire ≥10; (b). Generalized Anxiety Disorder Questionnaire ≥7); (c). positive response to 1 item panic attack screener; (d). evidence of suicidality. Patients were evaluated against the most common RCT exclusion criteria personality disorder, high suicide risk, cognitive impairment, psychosis, alcohol or substance abuse or dependency, bi-polar depression.

**Results:**

Total 81 HF patients were referred from 404 HF admissions, and 73 were assessed (age 60.6±13.4, 47.9% female). Nearly half (47%) met at least 1 RCT exclusion criterion, most commonly personality disorder (28.5%), alcohol/substance abuse (17.8%) and high suicide risk (11.0%). RCT ineligibility criteria was more frequent among patients with major depression (76.5% vs. 46.2%, p<.01) and dysthymia (26.5% vs. 7.7%, p = .03) but not significantly associated with anxiety disorders. RCT ineligible patients reported greater severity of depression (M = 16.6±5.0 vs. M = 12.9±7.2, p = .02) and were higher consumers of HF psychotherapy services (M = 11.5±4.7 vs. M = 8.5±4.8, p = .01).

**Conclusion:**

In this real-world sample comparable in size to recent RCT intervention arms, patients with depression disorders presented with complex psychiatric needs including comorbid personality disorders, alcohol/substance use and suicide risk. These findings suggest external validity of depression screening and RCTs could serve as a basis for level A guideline recommendations in cardiovascular diseases.

## Introduction

Depression has gained widespread research attention with respect to prognosis of heart diseases including heart failure (HF) [1]. A meta-analysis by Rutledge et al. [2] suggested that the prevalence of clinical depression was 22% in HF, thus substantially higher than community prevalence estimates for populations free from heart failure [3]. It has been consistently shown that depression doubles the risk of major cardiac events and death in patients with documented HF [2], [4], [5], increases healthcare costs [6], significantly impairs quality of life [7]–[9], impairs self-care ability [10] and impacts upon participation in HF disease-management strategies [11]. Consequently, depression identification and management is emphasized in international cardiology guidelines [12]–[15], HF treatment guidelines [16] and HF self-management recommendations [17].

Though a number of studies have applied routine depression screening protocols to improve recognition of depression [18]–[27] a paucity of information exists regarding the ensuing mental health management strategies initiated within cardiology settings from a positive depression-screen [22], [28]–[31]. Thombs and colleagues systematic reviews confirm that a number of issues regarding routine screening remain unclear [28], [29]. As the utility for depression screening alone in reducing depression and cardiovascular morbidity has not been established [22], randomised, controlled trials (RCTs) provide Level A empirical evidence to guide clinical practice for depression management in HF [2], [32]. For example, the Safety and Efficacy of Sertraline for Depression in Patients with Chronic Heart Failure trial was designed to facilitate easy translation into clinical practice [33]. Unfortunately, however, the extant depression RCT evidence in HF [32]–[36] has not been subjected to tests of external and ecological validity and therefore, the implications for clinical practice are not known [37]. Moreover, if external validity of depression RCTs is not established then unrealistic expectations regarding depression treatment response may be fostered among clinicians and patients alike [38]. Complicating these matters further, the American Heart Association guidelines recommend comprehensive assessment of other mental disorders such as anxiety [12] which are present in 30% of RCT patients with positive depression screen [39]. Yet Hasnain and colleagues [22] also emphasize the lack of guidance for individualized depression treatment plans when such comorbidity is present. Consequently, underestimation of the complexity of real-world mental health treatment needs may hamper concerted efforts to implement depression screening guidelines [12], [13], [16], [17] and integrate depression management into HF clinical practice [19], [40], [41].

The topical nature of routine depression and anxiety screening [28], [29] suggests it is timely to examine the practical implementation of integrated mental health care within real-world HF settings [30] subsequent to guideline based routine depression-screening initiatives. This study reports on referrals to a HF-specific psychologist generated from routine depression and anxiety screening in three public hospitals in Adelaide, South Australia. The following research questions will be answered:

1. To what extent are real-word HF-patients with depression covered by the inclusion and exclusion criteria of RCTs on depression in HF patients?
2. Do RCT ineligible patients differ from RCT eligible patients with respect to demographic and clinical characteristics?
3. What are the prevalence rates of various depression and anxiety disorders among HF patients referred for integrated mental health management after routine depression and anxiety screening?

## Methods

### Patient Selection

**Figure 1:**
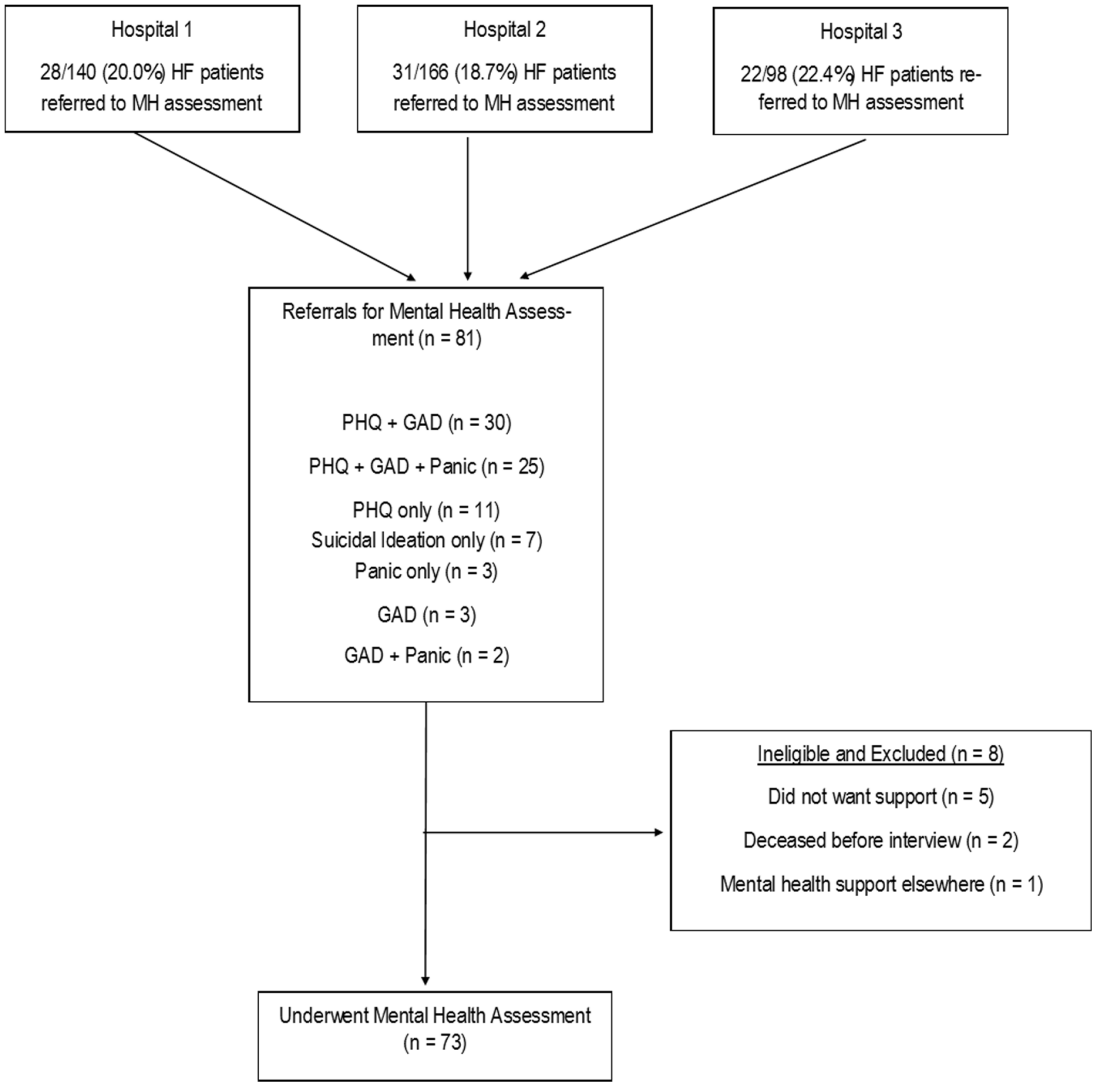
Flow chart of patients through the study.

This study received ethics approval and all participants provided written and informed consent prior to assessment (Human Research Ethics Committee of The Queen Elizabeth Hospital, Lyell McEwin Hospital and Royal Adelaide Hospital #HREC/12/TQEHLMH/188). Between April 2011 and June 2012 patients with verified HF admission were managed by specialist HF nurses in a HF self-management program (HFSMP) [17] at three South Australian hospitals (Queen Elizabeth Hospital, Royal Adelaide Hospital, Lyell McEwin Hospital). During this period specialist HF nurses routinely screened patients with validated questionnaires and referred patients to the HFSMP psychologist when either of the following criteria were met; (a). depression symptoms were in the clinically significant range (Patient Health Questionnaire (PHQ-9) ≥10); (b). anxiety symptoms were in the clinically significant range (Generalized Anxiety Disorder (GAD-7) ≥7); (c). patients had evidence of panic attack (N.B. questionnaires described further below). (d). there was evidence of suicidality (PHQ or identified by nurse). Median time between referral and assessment was 20 days. A flow chart of participants through the study is shown in Figure 1.

### HFSMP and Consent Process

Referred patients were contacted by telephone to schedule the initial mental health assessment and all facets of HFSMP care was provided at no cost. The HFSMP was community based, delivered flexibly at home visit, hospital site, or prior to weekly HFSMP exercise classes at Hampstead Rehabilitation Hospital. Ineligibility criteria for psychologist referral was not having cardiologist verified HF or currently receiving psychology and/or psychiatrist support elsewhere.

Patients who consented to standard HFSMP psychology assessment were free to refuse treatment at any time in accordance with ethical guidelines and government primary health care protocols. Patients not desiring the HFSMP psychology assessment (n = 5) were provided with alternative counselling arrangements including psychiatrist referral, local psychologist support and tele-counselling. Refusal did not impinge on standard cardiology care. HFSMP psychology was withdrawn in cases when patients transitioned to a palliative care team and the associated mental health supports. Patients requiring acute psychiatric care were managed by the treating psychologist in collaboration with the 24 hour South Australian Mental Health Emergency Triage Service for Community and Older Persons (Acute Crisis Intervention Service).

### Psychological Assessment

Referred patients repeated the depression and anxiety questionnaire at the psychologist intake assessment and again before each subsequent psychologist appointment to verify symptom response to treatment. The PHQ-9 [42] is a 9 item depression questionnaire covering major depression disorder criteria demarcated by DSM-IV [3]. Respondents endorse items based on the previous two-weeks on a scale of 0 “not at all” to 3 “nearly every day.” PHQ scores ≥10 warrant further evaluation according to the American Heart Association guidelines [12] and have favorable sensitivity and specificity for detection of depression disorder in heart disease populations [43].

Patients also completed an 8 item questionnaire regarding anxiety (Generalized Anxiety Disorder-7, 7 items (GAD-7); and a one-item panic screener “*In the last 4 weeks, have you had an anxiety attack – suddenly feeling fear or panic*?”) [44]–[46]. The GAD-7 is a 7 item anxiety questionnaire covering Generalized Anxiety Disorder criteria demarcated by DSM-IV [3] and GAD-7 scores ≥7 warrant further evaluation [46]. The single-item panic disorder screener also showed favorable sensitivity and specificity in detection of panic disorder amongst medical and psychosomatic medicine populations [47]. The anxiety measures were selected in addition to depression screening as the American Heart Association [12] guidelines advocate comprehensive assessment of anxiety disorders. Also, anxiety disorders are highly prevalent in heart diseases and predict higher rates of cardiovascular morbidity and mortality in patients with heart disease [48]–[54].

Patients were assessed with the Structured Clinical Interview for DSM-IV Axis-I and AXIS-II disorders [55], [56]. The SCID is a widely validated interview with favourable psychometric properties. Psychologist diagnoses were verified by two senior clinical psychologists once per month.

### Comparison with RCT Exclusion Criteria

Comparison of the present community treatment sample against RCT exclusion criteria focussed on depression interventions as there are no known anxiety disorder interventions in HF patients. Ineligibility for RCT was determined from the recent systematic review of depression interventions in HF reported by Woltz and colleagues [32]. Woltz et al. [32] evaluated 23 experimental and quasiexperimental HF trails (Total N = 3,564 patients) reporting depression symptom change from a range of interventions. Here we focussed solely on the six RCTs that evaluated either antidepressant or psychotherapy (i.e. cognitive-behavioural therapy, relaxation, mindfulness stress reduction) [33], [36], [57]–[61]. Ineligibility of our real world patients was determined against the six most common RCT exclusion criteria extracted from the included trials in Woltz et al [31]:

1. personality disorder [33], [36], [58], [61]: SCID diagnosis of a personality disorder
2. suicide risk [33], [36], [57], [59]; score of PHQ item 9≥2, verified at structured interview
3. cognitive impairment [33], [36], [57], [61]: Mini Mental State Examination ≤23, verified dementia or developmental disorder
4. current or past psychosis [33], [36], [57], [59], [61]: SCID diagnosis of a psychotic episode/disorder
5. active alcohol/substance abuse or dependency [33], [57], [59]–[61]: SCID diagnosis of alcohol/substance abuse or dependency
6. current or past bi-polar [33], [59], [61]: SCID diagnosis of bi-polar disorder

### Statistical Analyses

Data analysis was performed with SPSS® 19.0 (SPSS Inc., Chicago, IL). Descriptive comparisons between RCT eligible and ineligible groups employed the independent samples t-test, and the chi-square statistic with Fisher’s exact test as appropriate. All statistical tests were two-tailed, an alpha value p<.05 was considered statistically significant. This exploratory study pertains to RCT criteria validation and we have therefore not adjusted for multiple comparisons [62].

## Results

During the study period 81 patients were referred to HF mental health care, 8 were not included (did not want support (n = 5), HF death prior to mental health assessment (n = 2), receiving psychology treatment elsewhere (n = 1). This left a sample of 73 patients whom underwent mental health assessment and psychotherapy as appropriate (Flow Chart shown in Figure 1.).

### Prevalence of RCT Eligibility by Depression Criteria

**Figure 2:**
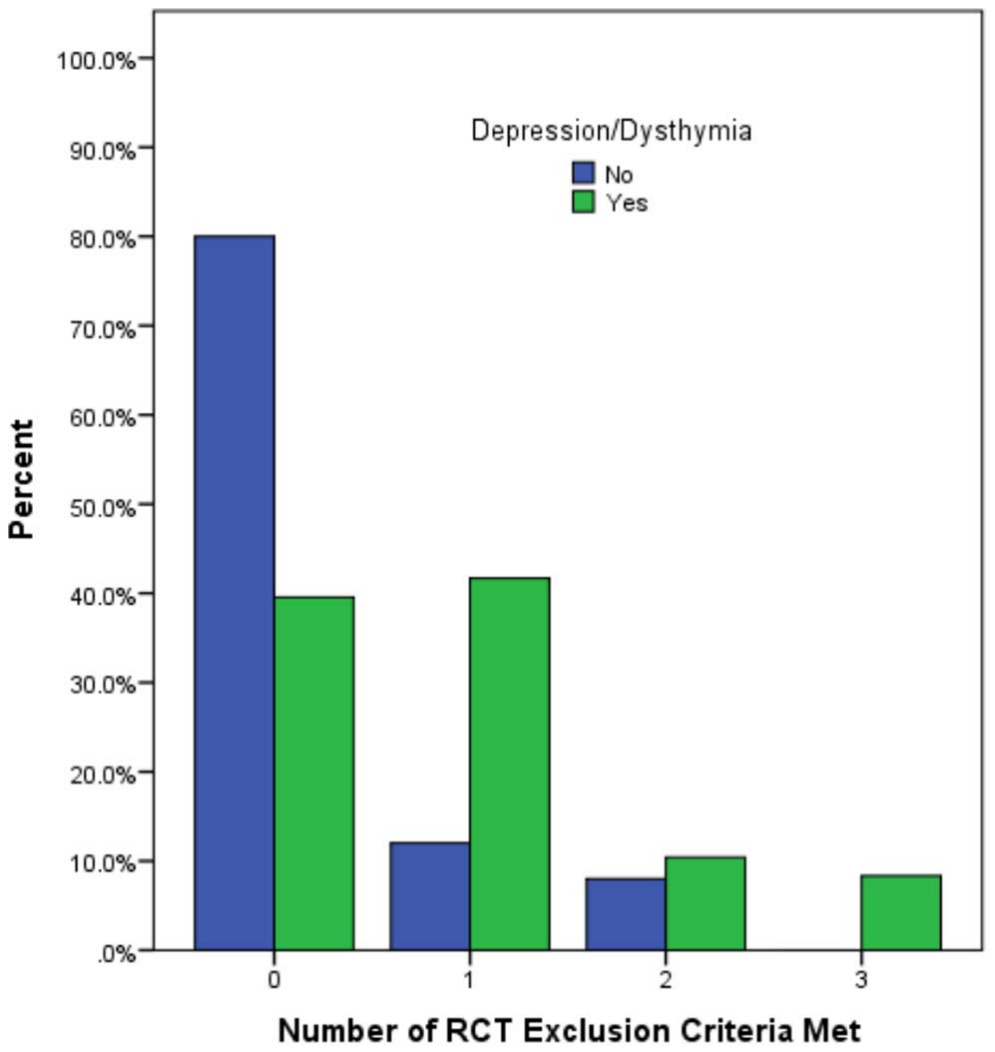
Proportion of standard RCT exclusion criteria met by HF patients according to depression status (yes vs. no).

Nearly half (46.6%) of assessed patients would be excluded from RCTs according to the six standard exclusion criteria (Table 1). The most common RCT exclusion criterions were personality disorder (28.8%), alcohol/substance abuse or dependency (17.8%) and suicide risk (11.0%). Analysis comparing the proportion of each RCT eligibility criteria between patients with depression disorder (major depression or dysthymia ± any anxiety) with those without depression disorder showed that patients with a mood disorder were more likely to meet at least 1 RCT exclusion criteria (60.4% vs. 29%, p<.001), particularly personality disorder (41.7% vs. 4.0%, p<.001) and alcohol/substance abuse or dependency (25.0% vs. 4.0%, p = .03). The proportion of RCT exclusion criteria by depression diagnosis is depicted in Figure 2.

**Table 1:** Psychiatric RCT ineligibility in heart failure patients referred for psychiatric care according to depression status.

*Psychiatric RCT exclusions*	Total N(%)N = 73	No Depression(N = 25)	Depression(N = 48)	P
Personality disorder	21 (28.8)	1 (4.0)	20 (41.7)	.001*
High suicide risk	8 (11.0)	3 (12.0)	5 (10.4)	1.0
Cognitive impairment	3 (4.1)	2 (8.0)	1 (2.1)	.27
Current or past psychosis	2 (2.7)	0	2 (4.2)	.54
Active alcohol/substance abuse or dependency	13 (17.8)	1 (4.0)	12 (25.0)	.03*
Current or past bi-polar	2 (2.7)	0	2 (4.2)	.54
Met any RCT exclusion criteria	34 (46.6)	5 (20.0)	29 (60.4)	.001*
Number of RCT exclusion criteria met				<.01*
1	23 (31.5)	3 (12.0)	20 (41.7)	
2	7 (9.6)	2 (8.0)	5 (10.4)	
3	4 (5.5)	0	4 (8.3)	

*RCT, randomized controlled trial.*

* *p<.05.*

### Demographic Descriptives According to RCT Eligibility

Comparison of the RCT eligible and ineligible patients with respect to demographics and comorbidities is shown in Table 2 (age M = 60.6±13.4, 47.9% female, 8.2% Indigenous Australian). RCT ineligible patients were more likely to have chronic pain documented in medical records (29.4% vs. 10.3%, p = .04). Ineligible patients were also less likely to have had a prior myocardial infarction (32.4% vs. 59.0%, p = .03), and there were no other significant differences.

**Table 2:** Comparison of RCT eligible and ineligible patients on demographics and medical comorbidity.

	Total N(%)N = 73	RCT EligibleN = 39	RCT IneligibleN = 34	P
*Demographic and comorbidity factors*				
Female	35 (47.9)	18 (46.2)	17 (50.0)	.74
Age, M(SD)	60.6 (13.4)	60.7 (13.9)	60.6 (13.1)	.98
Lives on own	19 (26.0)	8 (20.5)	11 (32.4)	.25
Disability Pension	22 (30.1)	11 (28.2)	11 (32.4)	.70
Indigenous Australian	6 (8.2)	4 (10.3)	2 (5.9)	.68
Current divorce/bereavement	8 (11.0)	3 (7.7)	5 (14.7)	.46
NYHA Class II	26 (35.6)	17 (43.6)	9 (26.5)	.35
III	39 (53.4)	18 (46.2)	39 (53.4)	
IV	8 (11.0)	4 (10.3)	8 (11.0)	
Left ventricular ejection fraction	33.9 (12.1)	34.2 (11.6)	33.5 (12.9)	.82
Prior myocardial infarction	34 (46.6)	23 (59.0)	11 (32.4)	.03*
Atrial fibrillation	25 (34.2)	11 (28.2)	14 (41.2)	.24
Coronary artery bypass	19 (26.0)	13 (33.3)	6 (17.6)	.13
Valve repair/replacement	12 (16.4)	4 (10.3)	8 (23.5)	.13
Biventricular pacemaker	11 (15.1)	7 (17.9)	4 (11.8)	.46
Implanted cardiac defibrillator	19 (26.0)	13 (33.3)	6 (17.6)	.13
Stroke/cerebrovascular accident	9 (12.3)	6 (15.4)	3 (8.8)	.40
Chronic obstructive pulmonary disease	22 (30.1)	11 (28.2)	11 (32.4)	.70
Renal disease	26 (35.6)	15 (38.5)	11 (32.4)	.59
Diabetes	38 (52.1)	21 (58.3)	17 (45.9)	.29
Hypertension	50 (68.5)	27 (75.0)	23 (62.2)	.24
Hypercholesterolemia	34 (46.6)	15 (41.7)	19 (51.4)	.41
Tobacco Smoking	31 (42.5)	18 (46.2)	13 (38.2)	.50
Body mass index kg/m^2^ >35	25 (24.2)	11 (28.2)	14 (41.2)	.24
Sleep apnea	13 (17.8)	5 (12.8)	8 (23.5)	.23
Chronic Pain	14 (9.2)	4 (10.3)	10 (29.4)	.04*

*NYHA, New York Heart Association; RCT, randomized controlled trial.*

* *p<.05.*

### Psychosocial Descriptives According to RCT Eligibility

Comparison of the RCT eligible and ineligible patients with respect to clinical psychiatric factors is shown in Table 3. RCT ineligible patients were more likely to receive anti-depressant treatment including amitriptyline (58.8% vs. 23.1%, p<.01) and reported greater severity of depression (PHQ M = 16.6±5.0 vs. 12.9±7.2, p = .02). RCT ineligible patients were also higher consumers of psychotherapy sessions during the HFSMP (M = 11.5±4.7 vs. 8.5±4.8, p = .01).

**Table 3:** Comparison of RCT eligible and ineligible patients on clinical psychiatric characteristics.

*Clinical Psychiatric Factors*	Total N(%) N = 73	RCT Eligible N = 39	RCT Ineligible N = 34	P
Psychotherapy sessions	9.8 (4.8)	8.5 (4.8)	11.5 (4.7)	.01*
Past suicide attempt	13 (17.8)	5 (12.8)	8 (23.5)	.23
No past psychiatric care	53 (72.6)	30 (76.9)	23 (67.6)	.44
Medical records depression	15 (20.5)	5 (12.8)	10 (29.4)	.08
Medical records missing depression diagnosis^a^	39 (53.4)	18 (46.2)	21 (61.8)	.18
Anti-depressant	29 (39.7)	9 (23.1)	20 (58.8)	<.01*
PHQ-9 total M(SD)	14.7 (6.5)	12.9 (7.2)	16.6 (5.0)	.01*
Any SCID anxiety disorder	52 (71.2)	26 (66.7)	26 (76.5)	.36
Medical records missing anxiety diagnosis^a^	42 (57.5)	21 (53.8)	21 (61.8)	.50
Current anxiolytic	21 (28.8)	8 (20.5)	13 (38.2)	.10
GAD-7 M(SD)	12.6 (6.7)	12.3 (7.1)	13.0 (6.4)	.67
Panic-Screener	28 (38.4)	13 (33.3)	15 (44.1)	.35

*GAD, Generalized Anxiety Disorder; PHQ, Patient Health Questionnaire; RCT, randomized controlled trial.*

* *p<.05.*

a *Medical records depression and medical records anxiety inclusive of diagnoses that conflicts with psychologist SCID assessment. Medical records missing depression or anxiety diagnosis evaluates medical record comorbidity lists and hospital discharge summaries in preceding 6 months prior to assessment with the SCID diagnosis.*

### Mental Health Prevalence Rates

The prevalence of depression and anxiety disorders is shown in Table 4 and stratified according to RCT eligibility. The most common disorders were major depression (60.3%), GAD (57.5%), panic disorder (52.1%) and social phobia (27.4%). Among the patients with a major depression or dysthymia diagnosis, 8/48 did not meet comorbid anxiety diagnosis. Comparison of patients based on RCT ineligibility showed an association with major depression (76.5% vs. 46.2%, p<.01) and dysthymia (26.5% vs. 7.7%, p = .03) and was not significantly associated with anxiety disorders.

**Table 4:** Prevalence of depression and anxiety disorders in heart failure patients referred for psychiatric care.

SCID Diagnosis	Total N(%) N = 73	RCT Eligible N = 39	RCT Ineligible N = 34	P
Major Depression	44 (60.3)	18 (46.2)	26 (76.5)	<.01*
Dysthymia	12 (16.4)	3 (7.7)	9 (26.5)	.03*
Panic +- agoraphobia	38 (52.1)	17 (43.6)	21 (61.8)	.12
Generalized anxiety disorder	42 (57.5)	19 (48.7)	23 (67.6)	.10
Post-traumatic stress disorder	14 (19.2)	5 (12.8)	9 (26.5)	.14
Obsessive-compulsive disorder	5 (6.8)	3 (7.7)	2 (5.9)	1.0
Social phobia	20 (27.4)	8 (20.5)	12 (35.3)	.16
Adjustment disorder	14 (19.2)	9 (25.0)	5 (13.5)	.21

*RCT, randomized controlled trial; SCID, Structured Clinical Interview.*

* *p<.05.*

## Discussion

This study reports the mental health status subsequent to depression and anxiety screening among HF patients. Psychological assessment suggested that patients commonly presented with emotional disorders other than depression including GAD and panic disorder, consistent with other research [54], [63]–[67]. However, psychiatric history would preclude nearly half of these HF patients from participation in contemporary depression RCTs based on six standard exclusion criteria identified from Woltz et al’s [32] systematic review. RCT ineligibility was highest amongst patients with depression disorders. Also, RCT ineligible patients reported greater severity of depression, chronic pain and were higher consumers of psychotherapy. Together the findings indicate that routine depression screening protocols may underestimate or not align with the real world psychiatric needs in HF. Consequently the extant depression treatment evidence may not even apply to half of cardiovascular patients referred for further psychiatric assessment.

A number of effective treatments for depression have been reported [68] though effects on suicidality are less clear [69]. The results here belie the assumption that depression is the only psychosocial factor for which HF patients require mental health care. These findings thus support the recent examples of routine anxiety screening in general cardiovascular patients [18], [64], [65]. Hasnain and colleagues [22] highlighted the absence of clinical guidance for individualized treatment plans when comorbid anxiety is present. The extent to which comorbid anxiety affects depression treatment response is unknown even though 30% of cardiac patients with a positive depression screen in a recent RCT met anxiety disorder criteria [39]. Clearly a limitation to mental health service provision and routine screening protocols among cardiovascular patients is the paucity of evidence-based treatments for individual anxiety disorders. Encouragingly, Shemesh et al showed that brief imaginal exposure and cognitive-behavioural therapy for PTSD after a cardiovascular event was associated with no marked increase in blood pressure, pulse and mean arterial pressure [70]. However safety of exposure-based anxiety treatments has not been demonstrated for GAD or panic [53]. These anxiety disorders were among the most common anxiety disorders prevalent here and elsewhere [54], [63] and may raise cardiovascular risk [71]. Nevertheless, psychotropic agents are utilized in early psychiatric intervention for anxiety disorders and also those psychiatric disorders which were RCT exclusions (i.e. alcohol/substance abuse, psychosis and bi-polar depression).

The current findings should not detract from the importance of prior RCT studies [32]–[36] and aspects of methodological rigour other than the six external validity criteria evaluated here. Application of appropriate exclusion criteria are essential to maintain internal validity. Other reasons justifying exclusion based on psychiatric criteria include ethical access to more appropriate treatment and reducing heterogeneity [72]. Diagnostic comorbidity also serves as a source of bias in depression treatment efficacy RCTs [73].

The current findings should thus serve to raise awareness regarding psychiatric illness complexity and comorbidity, particularly as treatment-resistant depression increases cardiovascular risk [74], [75]. Here, RCT ineligibility was primarily associated with depression disorders. Patients with mood disorder were significantly more likely to have personality disorders and active alcohol/substance abuse or dependency. Recently it was also documented that treatment seeking panic disorder patients also reported high rates of active alcohol/substance abuse and personality disorders [76]. The findings support the necessity of comorbidity assessment by qualified professionals after a positive depression screen [12]. Indeed, the clinical importance of such assessments are bolstered by findings that the functional aspects of HF do not correlate with suicide risk [77], whereas personality disorders, anxiety and depression are more established factors that increase suicide risk [78], [79].

The strength of this study was comprehensive psychological assessment after a routine depression and anxiety screening initiative in ambulatory HF patients thus facilitating mental health care tailored to individual patient needs. This study is presented with several limitations that temper the generalizability of these findings. Firstly, the use of anxiety questionnaires may have elicited more referrals for patients with comorbid anxiety-depression such as GAD and panic disorder [64], [65]. The referral of panic disorder in particular may correspond to the tendency to focus on dyspnea symptoms in HF treatment [80]. Secondly, ethical constraints precluded an evaluation of HF patients that were not routinely screened and/or not referred. Thus there was no comparison of the prevalence of those constituent variables for RCT eligibility in the general HF population. Reports also suggest approximately 27% of cases are not examined in international routine depression screening protocols [19]. Thirdly, it was not known whether there was a selection bias in referrals given the under-representation of patients with cognitive impairment [81]. Fourthly, the sample size, comparable or larger than most intervention arms in recent depression RCTs evaluated by Woltz et al [32], was potentially too small to draw broad conclusions regarding the psychiatric treatments needs and screening suggestions of HF patients generally. Fifthly, the pragmatic aspects of routine screening in HF need to be considered within the regional context by contrast to other cardiology settings and international experiences [18], [19], [22], [30], [64], [65], [82]. These findings from the current HFSMP may not generalise to other hospitals and it is unknown whether depression screening in conjunction with other management strategies in HF might beneficially impact depression remission rates. Finally, the potential for Type I errors is a limitation and as such will require confirmation in independent cohorts.

In conclusion, implementation of routine depression screening protocols in cardiology settings may underestimate the severity and complexity of psychiatric needs in HF such as comorbid personality disorders, alcohol/substance use, suicide risk and anxiety disorders. Application of six standard exclusion criteria suggested that the extant RCT evidence may not apply to half of HF patients referred for psychiatric care. Further investigation into external validity of depression RCTs in cardiology settings is recommended to better reflect typical HF patient needs [83]. These findings make the case for a specific focus on external validity of RCTs and depression screening protocols as basis for level A guideline recommendations.
